# A Retrospective Crossover Study on Cross-Linked Hyaluronic Acid Gel (Lacrifill) Versus Cyclosporine A 0.05% (Restasis) for Dry Eye Disease

**DOI:** 10.7759/cureus.91343

**Published:** 2025-08-31

**Authors:** Bradley A Nordin

**Affiliations:** 1 Ophthalmology, Huffman and Huffman Eye Physicians and Surgeons, London, USA

**Keywords:** canalicular plugs, cross-linked hyaluronic acid, dry eye disease, lacrifill, ocular surface disease index (osdi), private practice, restasis, retrospective crossover study, schirmer test, tear breakup time (tbut)

## Abstract

Purpose: This retrospective crossover study compares two-month outcomes of cyclosporine A ophthalmic emulsion 0.05% (Restasis) versus cross-linked hyaluronic acid gel (Lacrifill), a novel canalicular occlusive device, in 20 patients with refractory aqueous-deficient dry eye disease (DED) at a rural optometry clinic.

Methods: In patients with moderate to severe DED, two-month outcomes for Restasis and Lacrifill (after a washout period) were compared using tear breakup time (TBUT), Schirmer scores, corneal staining (Efron scale), and Ocular Surface Disease Index (OSDI) scores. Paired t-tests or Wilcoxon signed-rank tests were used (p<0.05), with strict adherence to ethical compliance.

Results: Both treatments improved TBUT, Schirmer scores, corneal staining, and OSDI from baseline (p<0.001). Lacrifill outperformed Restasis: TBUT (+2.28 vs. +1.30 s, p<0.001), Schirmer (+2.33 vs. +1.08 mm, p=0.001), staining (-1.03 vs. -0.55 grade, p=0.003), and OSDI (-33.33 vs. -10.83 points, p<0.001). Eye-level analyses (N=40) confirmed these findings, with no age or sex associations (p>0.05).

Conclusion: Lacrifill provided superior tear retention and symptom relief during the first two months of treatment compared to Restasis in refractory aqueous-deficient DED, supporting occlusion therapies in DED management. Larger prospective trials are needed to confirm efficacy and explore the use of combination therapies (typically three to six months).

## Introduction

Dry eye disease (DED) is a prevalent, multifactorial condition characterized by a disruption of tear film homeostasis, resulting in ocular discomfort, visual disturbance, and potential damage to the ocular surface [1]. Affecting a significant portion of the population, particularly older adults and individuals with autoimmune disorders, DED poses a substantial clinical challenge due to its diverse etiologies, including aqueous deficiency and evaporative mechanisms [1,2]. This study focuses on patients with the aqueous-deficient subtype, characterized by reduced tear production as evidenced by low Schirmer scores. Standard management strategies for DED typically follow a stepwise approach, beginning with artificial tears and progressing to anti-inflammatory therapies such as cyclosporine A 0.05% (Restasis) for moderate to severe cases [3,4]. Restasis, a topical immunomodulator, reduces ocular surface inflammation by inhibiting T-cell activation, a key driver in DED pathogenesis, thereby improving tear production [3,5]. However, a subset of patients experience inadequate symptom relief with Restasis, with meta-analyses indicating variable efficacy across formulations and potential tolerability issues such as burning or stinging upon instillation, necessitating alternative or adjunctive therapies to address persistent symptoms [6-8].

Lacrifill, a novel crosslinked hyaluronate canalicular gel occlusive device, offers a promising alternative by temporarily occluding the lacrimal canaliculi to enhance tear retention on the ocular surface [6,9]. Unlike traditional punctal plugs, Lacrifill’s gel-based delivery system offers a minimally invasive approach to improve tear film stability, potentially providing advantages in patient comfort, ease of administration, and a reduced risk of complications such as plug extrusion [6,10,11]. Lacrimal occlusion has been shown to effectively reduce DED symptoms and improve clinical signs, such as tear film stability and ocular surface health, making it a valuable strategy for patients with aqueous-deficient DED [12,13]. Recent studies, including patient satisfaction surveys, have highlighted the high tolerability of Lacrifill, with over 80% of users reporting no pain and minimal adverse events, positioning it as a viable option for refractory cases [6,14]. Despite its potential, limited clinical data exist on Lacrifill’s comparative effectiveness against established treatments like Restasis, particularly in non-responders, though analogous occlusion therapies have demonstrated comparable or superior outcomes in signs like tear breakup time (TBUT) in randomized trials [15,16]. Despite their differing mechanisms and onset times, Restasis's gradual anti-inflammatory action versus Lacrifill's rapid physical occlusion, this comparison targets a key unmet need in refractory DED, evaluating Lacrifill as an alternative for non-responders and exploring synergistic potential [3,6,13,16]. The purpose of this retrospective crossover study is to compare the results achieved after two months of treatment with Restasis versus Lacrifill in a cohort of 20 patients with aqueous-deficient DED at a rural optometry clinic who were non-responders to Restasis.

The within-subject design minimizes inter-individual variability, enhancing the study’s ability to detect differences in treatment effects within the same patients. By focusing on prior Restasis users, this study addresses a critical gap in the literature regarding the management of refractory DED in a rural clinical setting, where access to specialized care may be limited. The findings are expected to contribute valuable insights into the comparative efficacy of Lacrifill, potentially informing clinical decision-making and guiding future research into optimizing DED treatment strategies for patients with persistent symptoms.

## Materials and methods

This methods section details the design and execution of a retrospective crossover study comparing the effectiveness of Lacrifill (a crosslinked hyaluronate canalicular gel occlusive device) and Restasis (cyclosporine ophthalmic emulsion 0.05%) in managing DED at a rural optometry clinic. The study utilizes existing clinical data to evaluate treatment outcomes, focusing on patients who received both interventions sequentially. The methodology is structured to ensure reproducibility and alignment with standard clinical research practices, drawing from established protocols for DED assessment and statistical analysis [4,17].

Study design

**Figure 1:**
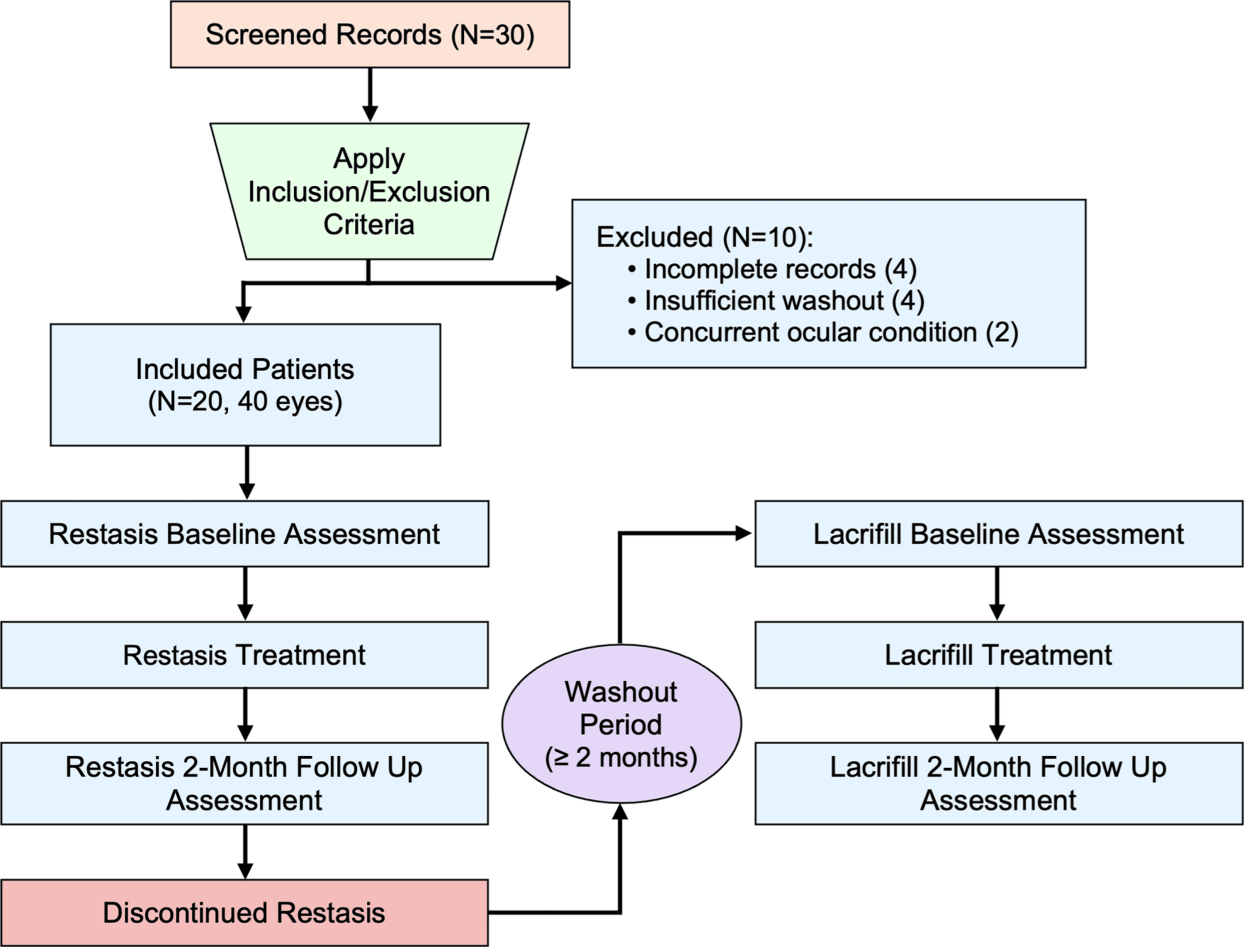
Flow diagram of retrospective crossover study design comparing Lacrifill and Restasis for DED DED, dry eye disease

This retrospective crossover study was conducted at a rural optometry clinic to compare the effectiveness of Lacrifill and Restasis in managing aqueous-deficient DED. The study employs a within-subject crossover design, where each patient serves as their own control, to evaluate clinical outcomes and test the hypothesis that Lacrifill will outperform Restasis in managing DED, with improvements comparable to those observed two months after traditional punctal plug placement. This hypothesis is based on Lacrifill’s mechanism of direct tear retention, which may more effectively address the limitations of anti-inflammatory therapies alone [13,16,18,19]. De-identified clinical records from patients treated since 2024 were analyzed. Each patient had sequentially received both treatments, with a minimum duration of two months for each intervention, allowing for a within-subject comparison. This design minimizes inter-individual variability, enhancing the ability to detect differences in treatment effects within the same patients [20,21]. Retrospective crossover designs are particularly useful in ophthalmology for evaluating sequential therapies in real-world settings, where randomization may not be feasible, as demonstrated in similar studies assessing serum eye drops for ocular surface disease [21,22]. Figure 1 illustrates the study design, including participant selection and treatment sequence.

Study population

Eligible participants were adults aged 47-74 years (mean 62.4±8.2 years) diagnosed with moderate to severe aqueous-deficient DED, defined by an OSDI score greater than 20 and a corneal staining score exceeding one on the Efron grading scale [4,23]. Patients must have completed at least two months of treatment with Restasis, followed by a two-month follow-up evaluation after starting therapy with Lacrifill. Exclusion criteria included ocular surgery within the past six months, use of other ocular medications (excluding artificial tears), or the presence of additional ocular conditions (e.g., glaucoma). Of the 30 patients initially identified as receiving treatment with Lacrifill at the practice, 20 met the inclusion criteria after excluding those with incomplete records or concurrent ocular conditions, which is consistent with sample sizes in similar DED studies [3,6]. The demographic characteristics of the study population consisted of 20 participants, with six males (30%) and 14 females (70%). The analysis encompassed 40 eyes in total, with 20 right eyes (oculus dexter, OD; 50%) and 20 left eyes (oculus sinister, OS; 50%).

Data collection

Data were extracted from electronic medical records at a rural optometry clinic, including demographics (age and sex), corneal staining scores (Efron grading scale), TBUT, Schirmer test results, and OSDI scores. Records of patients previously treated with Restasis prior to treatment with Lacrifill in 2025 were screened, and eligible data were entered into a secure database. All data were de-identified to ensure patient confidentiality, with birth and visit dates converted to age and treatment duration, respectively, and original dates discarded. This process ensured compliance with privacy regulations, such as HIPAA, and facilitated secure data handling for analysis [24].

Treatment details

Patients were instructed to administer Restasis (cyclosporine ophthalmic emulsion 0.05%) twice daily to the affected eye(s) for the duration of the treatment period, in accordance with standard clinical protocols [25]. Lacrifill, a cross-linked hyaluronic acid canalicular gel occlusive device, was administered according to the manufacturer’s guidelines [9], with clinical results measured at a two-month follow-up visit. The clinical course of each patient determined the sequence of treatments, with the switch to Lacrifill occurring due to inadequate response to Restasis (e.g., persistent symptoms, intolerable side effects, or administration challenges). After discontinuing Restasis, each patient completed a minimum two-month washout period before starting treatment with Lacrifill, during which patients returned to using warm compresses and artificial tears to control dry eye symptoms. Defining and ensuring the completion of an adequate washout period between treatments for all patients included in this study was essential to mitigate the residual effects of Restasis [17,26]. Comparable baseline measures across all study parameters (p>0.05, paired t-tests) confirm minimal residual effects from Restasis, as its immunomodulatory actions are reversible and typically wane within weeks of discontinuation [5,16,20]. Throughout the study, including the washout period, patients were permitted to use propylene glycol 0.6% ophthalmic emulsion (Systane Complete) artificial tears as needed for symptom relief, in accordance with clinic protocols and manufacturer instructions [9].

Outcome measures

Outcomes are assessed using standardized clinical measures, including TBUT to evaluate tear film stability, anesthetized Schirmer score to measure tear production, corneal staining grade on slit-lamp examination to assess ocular surface integrity, and the Ocular Surface Disease Index (OSDI) patient survey to capture patient-reported symptom severity [18,23]. The primary outcomes were the change in corneal staining score, TBUT (in seconds), anesthetized Schirmer test score (mm/5 min), and OSDI scores (0-100), providing a comprehensive evaluation of ocular surface health and patient-reported symptoms [4]. These measures provide a comprehensive evaluation of both objective and subjective aspects of DED, aligning with the recommendations of the Tear Film & Ocular Surface Society (TFOS) and Dry Eye Workshop (DEWS) II for assessing DED severity and treatment response, thereby ensuring consistency with established clinical standards [4,18].

Corneal staining assessment

Corneal staining was evaluated using the Efron grading scale, which quantifies the extent and severity of corneal epithelial damage on a 0-4 scale, with increments of 0.1 for finer assessment. This scale was chosen for its widespread use in clinical practice, its sensitivity in detecting changes in staining severity, and its validation for assessing ocular surface conditions such as those related to contact lens wear and DED [27,28]. The Efron grading scale is particularly suitable for retrospective studies, such as this one, where consistency across different time points and assessors is crucial. Its standardized, reproducible nature ensures that corneal staining assessments are reliable and comparable, even when conducted by different clinicians over time [27]. Furthermore, the scale’s ability to provide sensitive gradations (e.g., 0.1 increments) allows for the detection of subtle changes in corneal health, which is essential for evaluating the comparative efficacy of treatments like Lacrifill and Restasis in patients with refractory DED [28]. The consistent application of the Efron scale in optometry training curriculum, as well as by the trained optometrists of the practice that is the focus of this study, further enhances the reliability of the outcome measures.

Statistical analysis

Descriptive statistics were used to summarize demographic characteristics and outcome measures, with means, standard deviations, and ranges reported for continuous variables and frequencies for categorical variables. Given the crossover design, paired t-tests were used to compare changes in corneal staining scores, TBUT, Schirmer test results, and OSDI scores between the Restasis and Lacrifill treatment periods. Normality assessments (Shapiro-Wilk tests) confirmed that parametric tests (paired t-tests) were appropriate for most changes, except where Wilcoxon signed-rank tests were applied for non-normally distributed data (e.g., staining changes) [29]. A p-value less than 0.05 was considered statistically significant. Analyses were conducted at both eye-level (N=40) and patient-level (N=20). Patient-level data for TBUT, Schirmer test, and corneal staining scores were derived as bilateral averages for each patient to account for inter-eye correlations [20,21]. OSDI data are inherently patient-level, as questionnaires were completed per patient rather than per eye. Secondary analyses explored associations between outcomes and factors such as age, sex, and treatment sequence using regression models where feasible [20,30]. Stem-and-leaf plots and descriptive statistics revealed no major outliers that influenced the results, with robust M-estimators aligning closely with the sample means [31]. Data analysis was performed using IBM SPSS Statistics software (version 25), ensuring robust statistical handling of the crossover design [17].

Ethical considerations

The study protocol was reviewed by the Biomedical Research Alliance of New York (BRANY) Institutional Review Board and determined to be exempt from complete IRB oversight. All patient data were de-identified to protect confidentiality, and the study adhered to the principles of the Declaration of Helsinki, ensuring ethical conduct in the use of retrospective clinical data [32].

## Results

Following screening of de-identified records from a rural optometry clinic, a total of 20 patients (40 eyes) were included in the analysis. All participants had completed at least two months of Restasis treatment followed by two months of Lacrifill therapy, with complete data available for all outcome measures at baseline and endpoint for each phase. Patient demographics and baseline clinical characteristics (pre-Restasis) are summarized in Table 1. The cohort was predominantly female (70%), with a mean age of 62.4 years (SD 8.2). Baseline measures reflected moderate to severe DED, including mean OSDI scores exceeding 50, average corneal staining grades of 1.32, and mean Schirmer scores of 4.63 mm (indicating aqueous-deficient DED) [4,23]. Baseline values for the Lacrifill phase were comparable to those at the start of Restasis treatment (all p>0.05 via paired t-tests), confirming an adequate washout period and return of DED symptoms to pre-Restasis baselines, thereby supporting the study’s internal validity [5,20].

**Table 1:** Summary of pre-treatment clinical data for the study participants TBUT: tear breakup time, OSDI: Ocular Surface Disease Index patient survey score, 0-100

	Mean	Std. dev.	Minimum	Maximum
Age, y	62.4	±8.2	47	74
TBUT, s	4.83	±1.56	2	8
Schirmer test, mm	4.63	±1.59	1	8
Staining grade (Efron)	1.33	±0.77	0	3
OSDI score	57.55	±16.63	20.8	83.3

**Figure 2:**
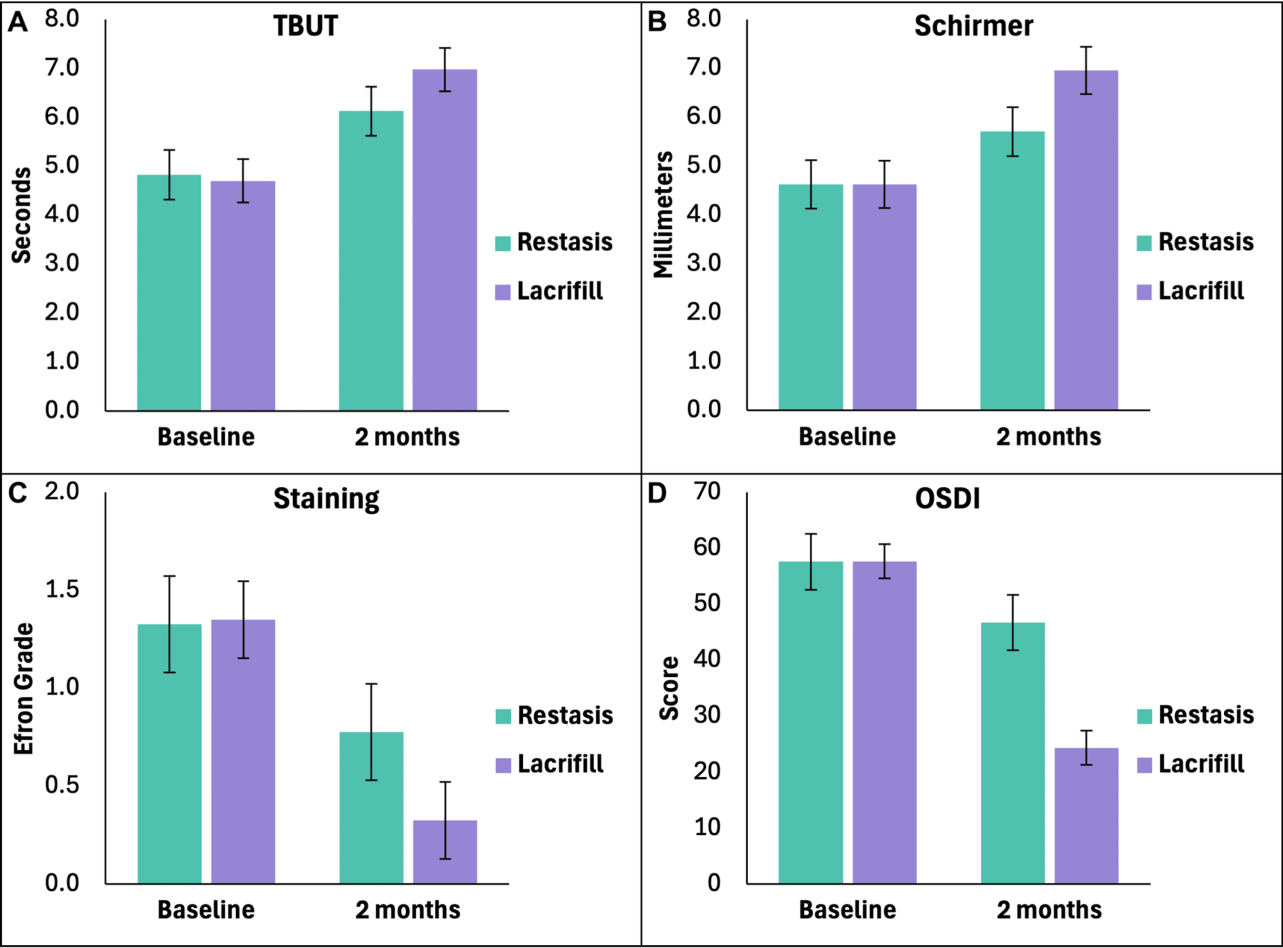
Changes in TBUT (A), Schirmer test (B), staining grade (C), and OSDI scores (D) for patients after two months of each treatment (N=20) Error bars represent 95% CI. Baseline means for each parameter showed no significant differences (p>0.05, paired t-test). All other differences were significant (p<0.05) by paired t-tests or Wilcoxon signed-rank tests, depending on data distribution. TBUT: tear breakup time, OSDI: Ocular Surface Disease Index patient survey score, 0-100

Both treatments yielded statistically significant improvements in all outcome measures from baseline to the two-month endpoint (all p<0.001). However, the magnitude of improvement was consistently greater with Lacrifill compared to Restasis. Changes in outcome measures per patient are summarized in Table 2 and illustrated in Figure 2.

**Table 2:** Changes in outcome measures after two months of each treatment (patient-level, N=20), with p-values from paired t-tests unless otherwise indicated ^a^Comparing change with Restasis to change with Lacrifill ^b^Wilcoxon signed-rank test for non-normal data distribution TBUT: tear breakup time, OSDI: Ocular Surface Disease Index patient survey score, 0-100

Outcome	Change with Restasis	p-value	Change with Lacrifill	p-value	Comparison p-value^a^
						TBUT, s	+1.30±0.71	<0.001	+2.28±0.94	<0.001	<0.001
Schirmer test, mm	+1.08±0.92	<0.001	+2.33±1.40	<0.001	0.001^b^
Staining grade (Efron)	-0.55±0.64	<0.001	-1.03±0.73	<0.001^b^	0.003^b^
OSDI score	-10.83±9.07	<0.001	-33.44±15.69	<0.001	<0.001

Direct within-subject comparisons of the changes showed that Lacrifill provided significantly greater improvements than Restasis in TBUT (p<0.001), Schirmer score (p=0.001), staining grade (p=0.003), and OSDI (p<0.001). Exploratory analyses at the eye level (N=40) yielded similar results, with all improvements being significant (p<0.001) and Lacrifill demonstrating superior changes (p<0.001 for all parameters). No significant associations were observed between treatment outcomes and patient age or sex (p>0.05 for all regression models) [30].

## Discussion

The findings of this retrospective crossover study indicate that Lacrifill, a crosslinked hyaluronate canalicular gel occlusive device, yields significantly greater improvements in both objective and subjective measures of aqueous-deficient DED compared to Restasis in patients who previously demonstrated inadequate response to the latter. Specifically, after two months of treatment, Lacrifill produced larger enhancements in TBUT, anesthetized Schirmer scores, corneal staining grades, and OSDI scores, with all differences achieving statistical significance (p≤0.003). These results support the hypothesis that Lacrifill's mechanism of direct tear retention through canalicular occlusion may be more effective in addressing DED in refractory cases, where anti-inflammatory therapies like Restasis are less effective [13,18]. These observed improvements correspond to clinically significant benchmarks in the DED literature, including OSDI score reductions of more than 12 points and Schirmer test increases exceeding 2 mm, both of which are linked to enhanced patient quality of life [3,18,23].

These findings align with the established pathophysiology of DED, characterized by tear film instability resulting from lacrimal gland dysfunction and evaporative loss [1,2]. Restasis, a topical cyclosporine A 0.05% emulsion, targets ocular surface inflammation by suppressing T-cell activation, yielding modest improvements in tear production and symptoms for many patients [3,5]. However, its efficacy varies, with meta-analyses reporting limited benefits and tolerability challenges, such as ocular stinging, contributing to non-response rates of 30-50% [7,8]. Notably, Restasis typically requires one to three months to show initial therapeutic effects and three to six months for maximal benefits in DED management [3,18]. This delayed onset likely explains the modest improvements observed at the two-month follow-up in this study, as patients, identified as non-responders, continued Restasis beyond this period before discontinuing it and switching to Lacrifill. While the two-month endpoint highlights Lacrifill's rapid benefits, it may underestimate Restasis's potential with prolonged use (three to six months) in non-responders, underscoring the need for longer-term studies to assess durability and combination approaches [3,18]. In contrast, Lacrifill’s superior outcomes at the same two-month mark underscore its capacity for rapid symptom relief, with occlusive effects lasting up to six months [6]. This comparison of two-month outcomes is a valuable contribution to the DED literature, highlighting Lacrifill’s efficacy in a timeframe where Restasis remains suboptimal, even for patients who persisted with it, thus guiding timely treatment decisions for refractory DED and potentially enhancing patient adherence and outcomes [3,18].

Lacrimal occlusion strategies, including Lacrifill, enhance tear retention directly, which has been shown to reduce symptoms and improve clinical signs such as TBUT and ocular surface integrity in aqueous-deficient DED [12,13]. Lacrifill's gel-based delivery offers advantages over traditional silicone or collagen punctal plugs, including reduced risk of extrusion, easier administration, and higher patient comfort, as evidenced by minimal adverse events and no reported pain in over 80% of users [6,10,14]. A recent multicenter trial further corroborated Lacrifill's efficacy, demonstrating sustained improvements in Schirmer scores and OSDI through six months, with noninferiority to hydrogel plugs [6,11].

The timeline of this study's two-month follow-up assessments highlights a compelling opportunity for combining Lacrifill with anti-inflammatory drops like Restasis from the outset of treatment, particularly in more severe or refractory cases, as the two modalities appear complementary in addressing the multifaceted nature of DED. Lacrifill's rapid onset of action provides immediate tear retention and symptom relief lasting up to six months [6], which aligns temporally with the three- to six-month period often required for cyclosporine-based therapies to achieve full therapeutic effectiveness in increasing tear production and reducing inflammation [33-37]. Moreover, the enhanced hydration of the tear film during this dual therapy period could promote ocular surface healing by mitigating hyperosmolarity and associated inflammation [37]. By initiating both simultaneously, patients could benefit from Lacrifill's short-term advantages to bridge the efficacy lag associated with Restasis, while the latter's long-term immunomodulatory effects sustain improvements once Lacrifill's temporary occlusion dissipates [13,18]. This approach is supported by evidence showing enhanced outcomes with combined punctal occlusion and cyclosporine, where plugs boost initial tear film stability and drop contact time, leading to superior symptom control compared to monotherapy [38,39]. Ultimately, such a strategy could mitigate key drawbacks, Lacrifill's finite duration and Restasis's delayed onset, potentially optimizing patient adherence and quality of life in refractory DED cases [3,16].

The superior performance of Lacrifill in this cohort is particularly noteworthy given the study's focus on Restasis non-responders, a subgroup often overlooked in DED research despite representing a significant clinical challenge [16,40]. Exploratory analyses revealed no significant associations between outcomes and demographic factors like age or sex, suggesting broad applicability across the study population, which was predominantly older and female, demographics commonly affected by DED [1,30]. These findings are consistent with analogous studies on punctal occlusion, where improvements in tear cytokine levels and symptom relief were comparable or superior to anti-inflammatory agents alone [13,19]. Moreover, the use of standardized assessments, including the Efron grading scale for corneal staining and TFOS DEWS II-recommended metrics, enhances the comparability of results to broader literature [4,27,28]. In rural optometry settings, where access to advanced therapies may be limited, Lacrifill's minimally invasive nature and high tolerability could facilitate better management of refractory DED, potentially reducing reliance on frequent topical instillations and improving adherence [9,11].

The within-subject crossover design represents a key strength, as it minimizes inter-individual variability and allows each patient to serve as their own control, which is particularly advantageous in retrospective analyses of sequential therapies [20,21]. This approach aligns with real-world clinical practice, where treatment switches occur based on response, and supports the internal validity through confirmed baseline equivalence post-washout [5,17,26]. Additionally, the robust statistical handling, including normality assessments and appropriate parametric/non-parametric tests, bolsters the reliability of the comparisons [29,31].

Limitations

Despite these strengths, several limitations must be acknowledged. The retrospective nature of the study introduces potential selection bias, as patients were not randomized, and treatment sequences were determined clinically rather than prospectively [22]. The small sample size (N=20 patients, 40 eyes) limits generalizability, particularly to diverse populations or milder DED cases, and may have resulted in underpowered subgroup analyses [3].

Additionally, results may not extend to evaporative or mixed subtypes of DED, as all patients in this cohort had aqueous-deficient DED. Furthermore, all corneal staining assessments were conducted by a single provider using the Efron grading scale, which, although promoting consistency in evaluation within this rural clinic setting, may introduce observer bias and preclude assessment of inter-rater reliability, a relevant consideration for subjective slit-lamp examinations in DED studies [27,28]. There was no blinding, which could influence subjective OSDI reporting. While a two-month washout mitigated carryover effects, residual influences from prior Restasis use cannot be entirely ruled out, particularly given the potential persistence of immunomodulatory effects on T cells (with lifespans up to three months in inflammatory settings) [16,17,20]. The two-month Restasis course and washout, although exceeding typical DED trial durations, may still favor Lacrifill's rapid effects, potentially leading to false-positive interpretations of superiority. However, baseline equivalence post-washout supports the validity of these comparisons.

Finally, data were derived from a single rural clinic, potentially reflecting site-specific practices, and long-term outcomes beyond two months were not evaluated, which limits insights into sustained efficacy and highlights the need for future prospective trials with at least six-month durations and larger cohorts [24].

## Conclusions

This study provides preliminary evidence that Lacrifill outperforms Restasis during the first two months of treatment in managing refractory DED, offering a promising alternative for non-responders through enhanced tear retention and symptom relief. These results underscore the value of occlusion therapies in stepwise DED management and warrant larger, prospective randomized trials to confirm efficacy, assess durability, and explore combinations with anti-inflammatory agents. Future updates or companion studies with extended follow-up could further elucidate durability, potentially as post-publication contributions to refine DED management strategies. Ultimately, integrating Lacrifill into clinical protocols could improve outcomes for patients with persistent DED, particularly in underserved settings, either as an alternative for non-responders or in dual therapy combinations to leverage their synergistic mechanisms.
